# Visual discrimination of screen-detected persistent from transient subsolid nodules: An observer study

**DOI:** 10.1371/journal.pone.0191874

**Published:** 2018-02-13

**Authors:** Kaman Chung, Francesco Ciompi, Ernst T. Scholten, Jin Mo Goo, Mathias Prokop, Colin Jacobs, Bram van Ginneken, Cornelia M. Schaefer-Prokop

**Affiliations:** 1 Department of Radiology and Nuclear Medicine, Radboud University Medical Center, Nijmegen, the Netherlands; 2 Department of Radiology, Seoul National University College of Medicine, and Institute of Radiation Medicine, Seoul National University Medical Research Center, Seoul, Korea; 3 Department of Radiology, Meander Medical Center, Amersfoort, the Netherlands; Northwestern University Feinberg School of Medicine, UNITED STATES

## Abstract

**Purpose:**

To evaluate whether, and to which extent, experienced radiologists are able to visually correctly differentiate transient from persistent subsolid nodules from a single CT examination alone and to determine CT morphological features to make this differentiation.

**Materials and methods:**

We selected 86 transient and 135 persistent subsolid nodules from the National Lung Screening Trial (NLST) database. Four experienced radiologists visually assessed a predefined list of morphological features and gave a final judgment on a continuous scale (0–100). To assess observer performance, area under the receiver operating characteristic (ROC) curve was calculated. Statistical differences of morphological features between transient and persistent lesions were calculated using Chi-square. Inter-observer agreement of morphological features was evaluated by percentage agreement.

**Results:**

Forty-nine lesions were excluded by at least 2 observers, leaving 172 lesions for analysis. On average observers were able to differentiate transient from persistent subsolid nodules ≥ 10 mm with an area under the curve of 0.75 (95% CI 0.67–0.82). Nodule type, lesion margin, presence of a well-defined border, and pleural retraction showed significant differences between transient and persistent lesions in two observers. Average pair-wise percentage agreement for these features was 81%, 64%, 47% and 89% respectively. Agreement for other morphological features varied from 53% to 95%.

**Conclusion:**

The visual capacity of experienced radiologists to differentiate persistent and transient subsolid nodules is moderate in subsolid nodules larger than 10 mm. Performance of the visual assessment of CT morphology alone is not sufficient to generally abandon a short-term follow-up for subsolid nodules.

## Introduction

Results of lung cancer screening Computed Tomography (CT) studies revealed the importance of subsolid nodules as potential early adenocarcinomas. In the Early Lung Cancer Action Project (ELCAP) study the prevalence of subsolid nodules was found to be lower compared to solid nodules. However, subsolid nodules demonstrated a higher malignancy rate in the detected subsolid nodules of 34% (15/44) compared to 7% (14/189) for solid nodules [1]. Another study evaluating a group of clinically and screen-detected lesions even reported 81% (43/53) of resected subsolid nodules to be (pre)malignant [2].

The most frequent benign disease causing subsolid nodules is a focal infection [3, 4]. Other more rare underlying benign diseases are a focal organizing pneumonia or focal fibrosis [5, 6]. Subsolid nodules caused by infection will eventually disappear. Differentiation of transience versus persistence of subsolid nodules thus represents the first diagnostic task to discriminate between benign and potentially malignant lesions, and a short-term three months follow-up has been recommended by the Fleischner Society and the British Thoracic Society [7, 8]. The percentage of subsolid nodules detected in screening studies varied from 2% to 20% of all baseline screen-detected non-calcified nodules [1, 9, 10]. Prospective discrimination of transient from persistent lesions would therefore contribute to the reduction of follow-up CTs. Previous studies on this subject evaluated the contribution of texture analysis and clinical features, but did not assess human observer performance [11–13].

The only other morphological feature used for risk prediction of subsolid nodules besides persistence and lesion growth, is nodule size and the presence/size of a solid component [10, 14]. For solid nodules spiculation is an important predictor of malignancy in a recently published (screening) risk model [10]. However, for subsolid nodules no additional morphological features have been established. Defining morphological features for transient and persistent subsolid nodules would be a valuable first step.

The purpose of this study was therefore to evaluate whether and to which extent experienced radiologists would be able to differentiate transient from persistent subsolid nodules from a single CT examination by visual analysis alone. Secondly, we aimed to identify which morphological features are used by the radiologists to make this differentiation.

## Materials and methods

### Study population

We recruited subsolid nodules from the National Lung Screening Trial (NLST). The NLST was approved by the institutional board at each participating medical institution and participants provided written informed consent before randomization [15]. In total the NLST had 26,722 participants. Of those, 3194 participants had at least one subsolid nodule annotated by the NLST screening radiologist in any of the 3 screening rounds. Nine participants did not have any scans available, leaving 3185 participants for further analysis.

For this observer study, we used baseline (year 0) subsolid nodules only. The NLST annotations did not contain year-to-year linking between the same lesions, therefore we re-annotated all lesions by using information from the NLST database (slice number, nodule type, lobe location, size). Annotations were done by two medical students and one medical researcher using in-house software (CIRRUS Lung Screening, Diagnostic Image Analysis Group, Radboud University Medical Center, Nijmegen, the Netherlands). A subsolid nodule was defined as transient if the nodule had disappeared on follow-up CT. A subsolid nodule was defined as persistent if the nodule remained visible on follow-up CT.

Subsequently we only selected CTs with a slice thickness of ≤ 2 mm, to ensure the quality of the coronal and sagittal projections of the lesions. As morphology is more difficult to assess in smaller lesions and thicker slices, we only selected lesions ≥ 10 mm (rounded average diameter) in this observer study. In total 232 subsolid lesions were eligible for our study. Eleven lesions (11/232, 4.7%) could not be located on the scans. Thus, our final dataset for the observer study contained 221 subsolid lesions.

### Observer study

All study lesions were independently evaluated by four experienced radiologists (ETS, CSP, MP, and JMG). All of them had > 15 years of experience in reading chest CTs and had extensive experience with evaluating screen-detected nodules. Nodules were presented in a random order to each observer. Observers were asked to score the morphological nodule features using a predefined list. The list of morphological features as well as the definitions can be found in Table 1. In addition, they were asked to estimate the probability that the lesion was persistent on a scale between 0 and 100, with 0 representing certainly transient and 100 representing certainly persistent.

**Table 1:** List of morphological features scored by each observer. For all features one category had to be chosen obligatorily.

Feature	Possible categories	Definition
Nodule type	- non-solid - part-solid - other	*Non-solid*: hazy increased attenuation in the lung that does not obliterate the bronchial and vascular margins^#^ *Part-solid*: consists of both ground-glass and solid soft-tissue attenuation components^#^ *Other*: any other nodule type that is not a subsolid nodule (e.g. solid nodule, calcified nodule) or pseudo nodule (“mimics a pulmonary nodule”)^#^ ^**#**^ definitions from the Fleischner Society: glossary of terms for thoracic imaging [22]
Nodule multiplicity	- solitary - multiple	Multiple nodular opacifications organized as a group within the same lobe
Lesion margin whole lesion	- ill-defined well-defined *if well-defined*, *specify (only one option possible)*: - linear demarcation - lobulated - spiculated - smooth	*Linear demarcation*: following the lobular border *Lobulated*: undulated contour of the border *Spiculated*: with lines radiating from the borders *Smooth*: a well-defined border which is not lobulated, spiculated or linearly demarcated
Solid core margin	- ill-defined - well-defined *if well-defined specify (only one option possible*: - linearly demarcated - lobulated - spiculated - smooth - multifocal	*Multifocal*: multiple spots of the solid core
Density of the ground glass component	- low - high	*Low*: faintly visible *High*: substantially higher than lung parenchyma but still fulfilling the criteria of ground-glass
Aspect of the ground-glass component	- homogeneous - inhomogeneous	With respect to density distribution
Air bronchogram	- no - solitary - multiple	Tubular air inclusions
Bubble lucency	- no - yes	Non-tubular air inclusions larger than neighboring bronchial structures
Pleural retraction	- no - yes	Displacement of the interlobar fissure or pleura
External retraction of the lung parenchyma	- no - yes	Distortion of the parenchymal architecture. This can be intranodular or extranodular, indicated by distortion of vessels or airways (signs of traction, displacement of neighboring bronchovascular structures).

### Reading methodology

**Fig 1:**
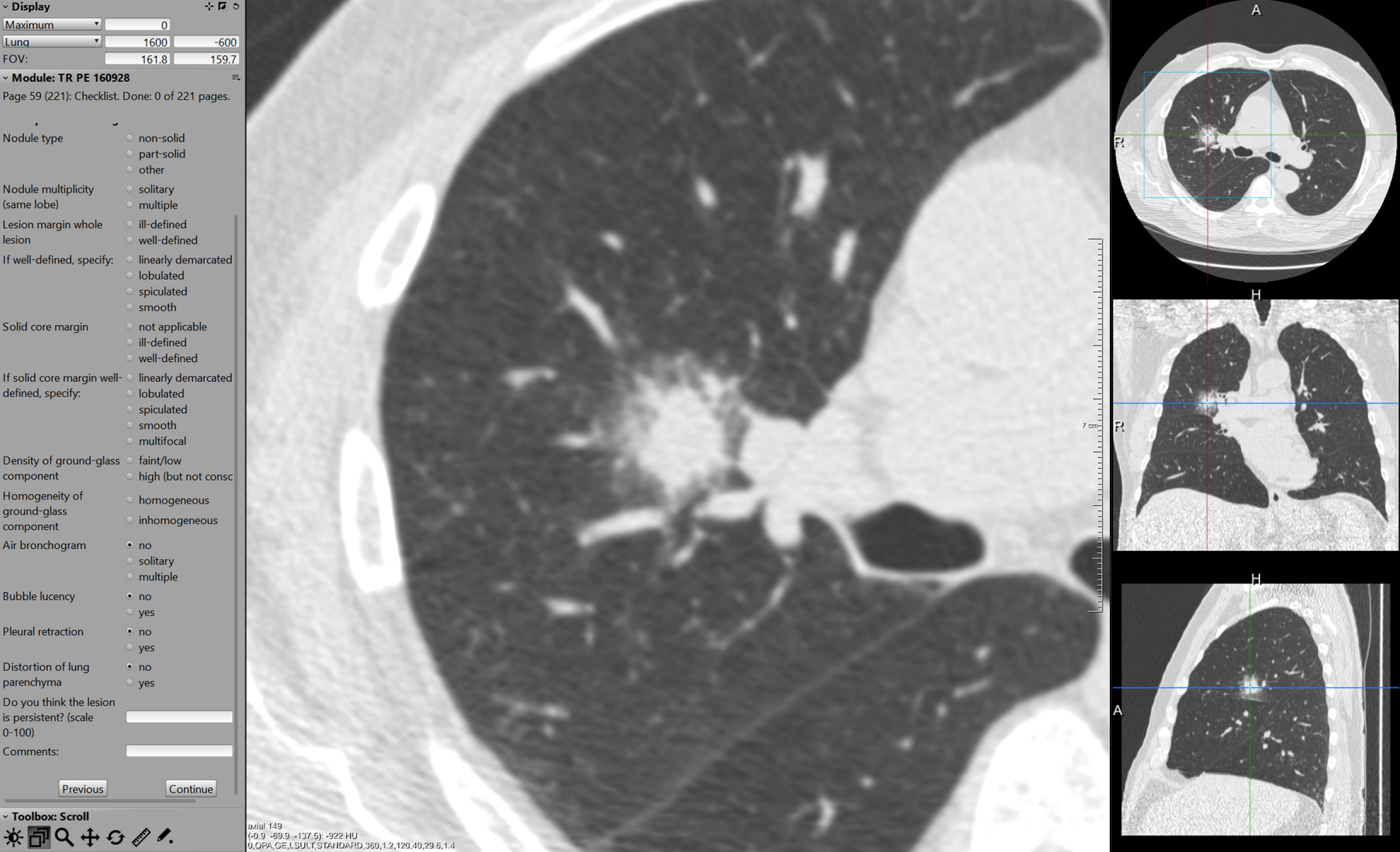
Reading workstation. The morphological features to be scored are listed on the left side of the monitor display. Lower-left corner has two text fields to enter the probability (0–100) and any comments. A magnified axial view of the nodule under evaluation is centered in the middle of the display. Coronal/sagittal projections are available on the right side of the screen, display size of the three projections was interchangeable. Processing tools such as windowing and magnification as well the full 3D CT dataset were available at any time.

A reading workstation designed to optimize workflow and to document the scoring data was used (CIRRUS Observer, Diagnostic Image Analysis Group, Radboud University Medical Center, Nijmegen, the Netherlands). After opening a case, a magnified axial view of the nodule under evaluation was centered in the middle of the display. Coronal/sagittal projections were available on the right side of the screen (Fig 1). The position of the magnified view was indicated by center lines on the smaller views. Using this set-up, observers did not have to search for the lesion. For all cases, the full 3D CT dataset was available for evaluation. All views could be (de)magnified. A standard lung window with a width of 1500 HU and a center of -650 HU was used as a default, but could be adjusted if necessary. Nodule diameters could be measured manually using electronic calipers.

The morphological features to be scored were listed on the left side of the monitor display. Scoring had to be completed before the next lesion could be displayed. Observers were allowed to place any comments if needed. No specifications with respect to comments were made prospectively. Lesions indicated in the comments for exclusion by 2 or more observers were omitted for further analysis. No information about follow-up appearance, persistence or any other outcome was provided.

### Statistical analysis

Receiver Operating Characteristic (ROC) analysis was performed for each observer. Areas under the curve (A_z_) and 95% confidence intervals (CI) were calculated to determine the ability to differentiate between transient and persistent lesions. We did not take into account within-participant correlation of participants with multiple nodules, because multiple nodules in a participant were considered as multiple independent nodules [16, 17]. Univariate analysis (Chi-square) per observer was used to assess whether a certain morphological feature was scored significantly different in transient or persistent nodules. P-values < 0.05 were considered significant. Inter-observer agreement for each CT morphological feature was investigated by calculating percentage agreement for each pair of observers. Statistical analyses were performed using SPSS, version 20.0 (SPSS, Chicago, Ill).

## Results

### Study group

Of all lesions 61% (135/221) were persistent. The median average diameter of persistent lesions was 12.0 mm (IQR 10.0–15.0 mm). Accordingly, 39% (86/221) were transient and had a median average diameter of 12.0 mm (IQR 11.0–16.5 mm). Forty-nine lesions were excluded from further data analysis because at least 2 of the 4 observers had made the comment that the opacification under review, which had been marked as subsolid nodule in the NLST database, would in fact not represent a nodular (subsolid) opacification when taking all three planes into consideration. Comments leading to exclusion were non-nodular (N = 11), solid lesion (N = 8), wall of emphysema (N = 3), apical scarring, (N = 7), fibrosis (N = 5) and (plate-like) atelectasis (N = 15). Thus the final study group consisted of 172 subsolid lesions (101 persistent, 71 transient).

### Discrimination of persistent from transient nodules

**Fig 2:**
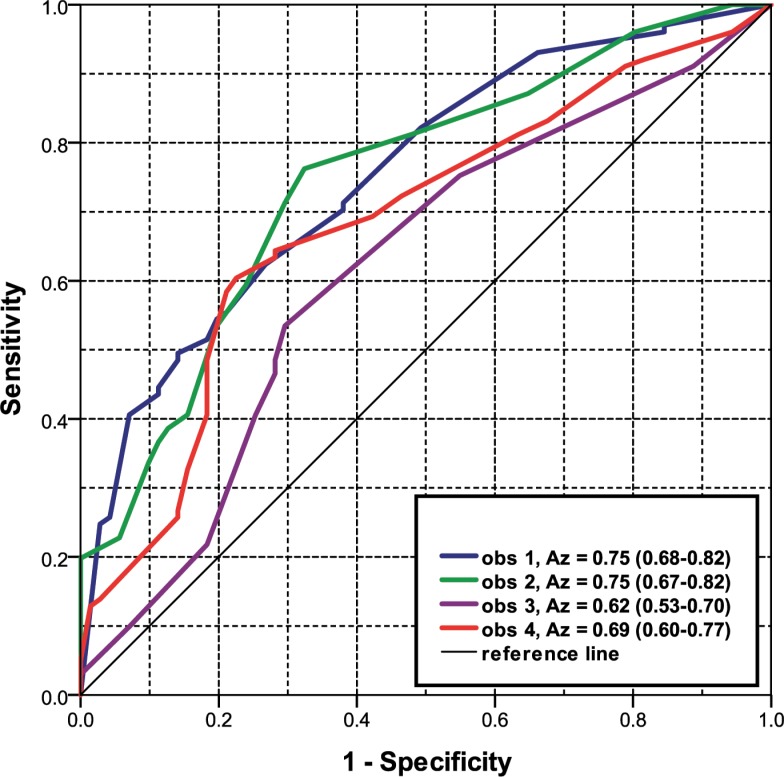
Receiver Operating Characteristic (ROC) curves for observer 1, 2, 3 and 4 to predict the persistence of the subsolid lesions ≥ 10 mm. A_z_ (Areas Under the Curve) and 95% confidence interval in parenthesis, obs = observer.

Observers 1 to 4 separately achieved an A_z_ for discriminating persistent from transient subsolid nodules of 0.75 (95% CI 0.68–0.82), 0.75 (95% CI 0.67–0.82), 0.62 (95% CI 0.53–0.70) and 0.69 (95% CI 0.60–0.77), respectively (Fig 2).

Considering the score of 50 as a threshold for discriminating between transience (scores 0–50) and persistence (scores 51–100), the four observers correctly identified 58/71 (82%), 63/71 (89%), 51/71 (72%) and 55/71 (77%) transient lesions. The observers correctly identified 52/101 (51%), 37/101 (37%), 47/101 (47%) and 61/101 (60%) persistent nodules, respectively.

**Fig 3:**
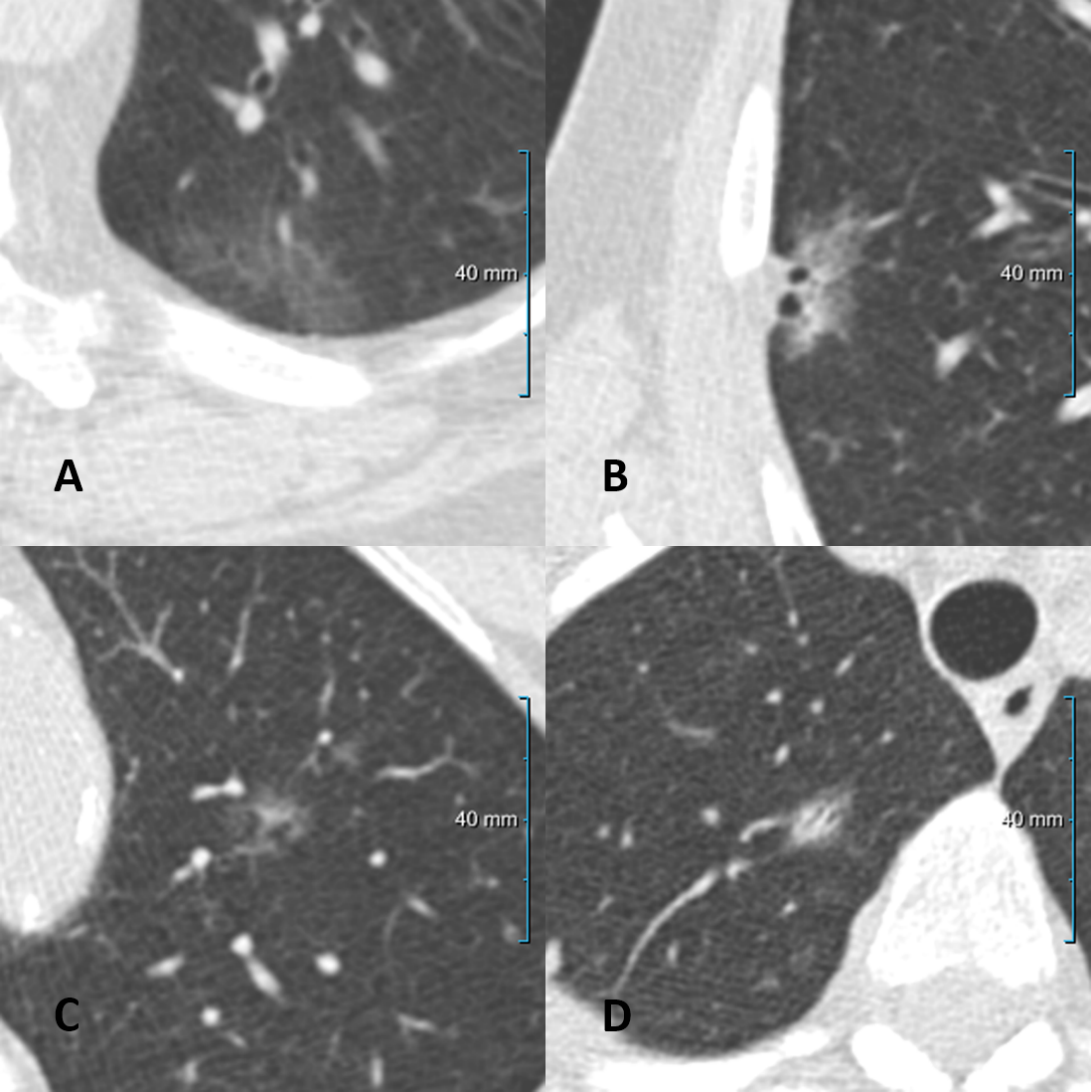
**(a)** Correctly identified transient lesion with a probability score of ≤ 40 by all four observers. **(b)** Correctly identified persistent lesion with a probability score of ≥ 80 by all four observers. **(c)** Incorrectly identified lesion by majority of observers: transient lesion, but scored as persistent (probability score ≥ 60). **(d)** Incorrectly identified lesion by majority of observers: persistent lesion, but scored as transient (probability score ≤ 40).

**Fig 4:**
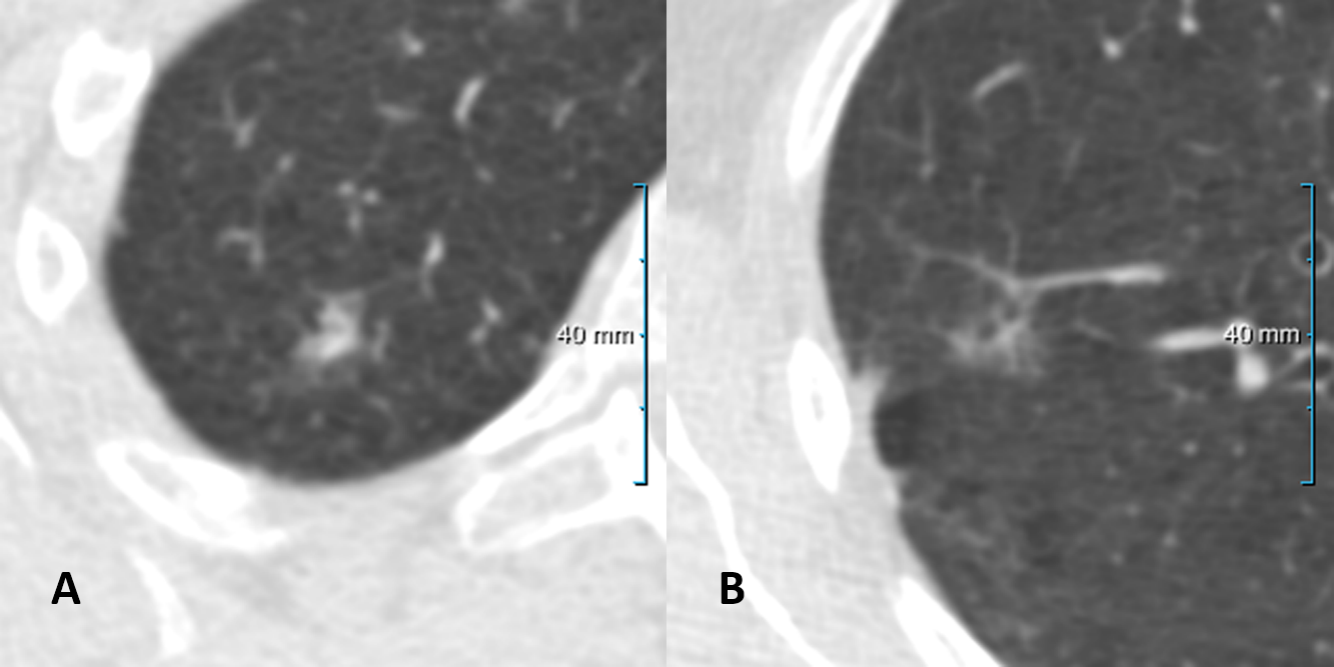
**(a)** A transient lesion with disagreement (2 versus 2) among observers. **(b)** A persistent lesion with disagreement (2 versus 2) among observers.

Taking the same thresholds for transience (0–50) and persistence (51–100), all four observers agreed on the same classification in 105 of the 172 nodules (61%). 68 of these 105 nodules (65%) were correctly classified, 37 of the 105 nodules (35%) were misclassified by all four observers. Thirty of the correctly classified nodules were persistent and 38 were transient. Figs 3 and 4 show examples of correctly and incorrectly identified lesions for which all or the majority of observers agreed on the classification.

**Fig 5:**
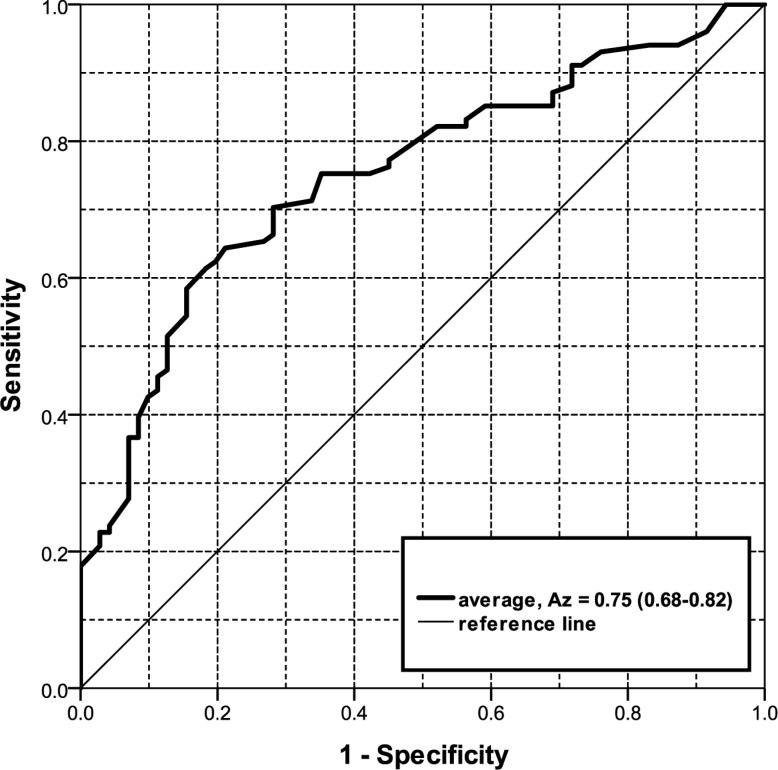
Receiver Operating Characteristic (ROC) curves for the average of all four observers. A_z_ (Area Under the Curve) and 95% confidence interval in parenthesis.

Averaging the scores of the four observers resulted in an A_z_ of 0.75 (95% CI 0.68–0.82) (Fig 5). Using the average scores a sensitivity of > 90% for persistent lesions was only achieved at the expense of a specificity of < 30% (e.g., sensitivity/specificity is 91% / 28%).

### Morphology assessment: Univariate analysis

Morphological features that showed significant difference between transient and persistent in at least 2 observers are listed in Table 2. At a significance level of p < 0.05, nodule type and lesion margin were scored significantly different by 2 observers (p = 0.016 and p = 0.025, p = 0.001 and p = 0.044 respectively). Part-solid nodules were more often seen in persistent lesions compared to transient lesions in all observers, reaching statistical significance in two of them (p = 0.016 and p = 0.025). The subcategory of a well-defined border yielded significant difference in 2 observers (p < 0.001 and p = 0.001). Linear demarcation following the lobular border was the only feature in this category to be seen more often in transient lesions in three observers. Lobulated, spiculated and smooth borders were scored more often in persistent lesions. Pleural retraction was observed more frequently in persistent than transient lesions reaching significance in two observers (p = 0.006, p = 0.037).

**Table 2:** Univariate analyses. Table describes morphological features with at least 2 observers in which the feature is seen significantly different between transient (T) and persistent (P) subsolid nodules using Chi-square. The total number of included nodules after exclusion is 172.

	**T**	**P**	**P-value** **Obs. 1**	**T**	**P**	**P-value** **Obs. 2**	**T**	**P**	**P-value** **Obs. 3**	**T**	**P**	**P-Value** **Obs. 4**
**Nodule type**												
non-solid	46	49		45	47		43	43		49	49	
part-solid	23	52	**P = 0.016**	25	53	P = 0.081	27	55	P = 0.064	21	48	**P = 0.025**
other	2	0		1	1		1	3		1	4	
**Lesion Margin**												
ill-defined	29	30	P = 0.130	55	51	**P < 0.001**	37	68	**P = 0.044**	38	39	P = 0.053
well-defined	30	71		16	50		34	33		33	62	
**If well-defined**	**N = 112**			**N = 66**			**N = 67**			**N = 96**		
linearly demarcated	27	13		1	3		18	9		23	16	
lobulated	1	15	**P < 0.001**	8	28	P = 0.813	0	1	P = 0.063	7	20	**P = 0.001**
spiculated	5	14		1	4		0	2		3	16	
smooth	8	29		7	14		15	22		1	10	
**Pleural Retraction**												
no	66	78	**P = 0.006**	71	96	P = 0.057	67	95	P = 0.933	68	87	**P = 0.037**
yes	5	23		0	5		4	6		3	14	

### Inter-reader variability of morphology

Average pair-wise percentage agreement was highest in external retraction, pleural retraction and bubble lucency (95%, 89%, and 86% respectively). Nodule type had an agreement of 81%, followed by nodule multiplicity (73%), solid core margin (71%) and presence of an air-bronchogram (70%). Lower agreement was found in density of ground-glass component (67%), lesion margin (64%) and the subcategory of a well-defined solid core margin (62%). Lowest agreement was found for aspect of ground-glass component (53%) and the subcategory of a well-defined lesion margin (47%). The average pair-wise agreement and the 95% confidence intervals can be found in Table 3.

**Table 3:** Average pair-wise percentage agreement of the morphological features.

Morphological feature	Average percentage agreement	95% Confidence Interval
Nodule type	81%	79–84
Nodule multiplicity	73%	61–84
Lesion margin	64%	56–71
Subcategory of well-defined margins	47%	44–50
Solid core margin	71%	67–75
Subcategory of well-defined solid core margins	62%	59–65
Density of ground-glass component	67%	64–69
Aspect of ground-glass component	53%	45–61
Air bronchogram	70%	59–82
Bubble lucency	86%	82–89
Pleural retraction	89%	86–92
External retraction	95%	92–98

## Discussion

The most frequent cause of transient subsolid nodules is focal infection. A persistent subsolid nodule, however, is potentially malignant and requires follow-up or alternative diagnostic work-up. A prospective estimation of whether the lesion would be persistent or transient would aid in reducing unnecessary follow-ups. This is the first study assessing the performance of human visual analysis for predicting the likelihood of persistence in subsolid nodules. Results of our study indicate that experienced radiologists are at best only moderately able (average A_z_ of all readings 0.75) to visually differentiate transient from persistent character in subsolid nodules ≥ 10 mm. In addition the individual performance among the observers varied substantially with A_z_ values ranging from 0.62 to 0.75. Given the variability among the observers, the moderate agreement and the imperfect performance of experienced radiologists, human visual analysis alone has to be considered insufficient to reproducibly predict if a subsolid nodule is persistent or transient. In that respect our results confirm published management strategies [7, 8] that recommend a 3-month follow-up CT for clarification of persistency.

A study by Lee HJ et al. [18] evaluated the performance of radiologists predicting benign and malignant subsolid nodules, a differentiation that might be less complex, since persistent lesions can be both benign and malignant and malignant lesions may expose more suggestive features. However, even with the availability of several clinical parameters (age, sex, pack years, history of lung cancer) and knowledge of predefined predictive CT information, an average A_z_ value of 0.77 for non-solid and of 0.76 for part-solid nodules were achieved, thus in fact comparable to our results.

Secondly, we found that none of the morphology features yielded significant discrimination in all four observers. Most promising features were nodule type, lesion margin, presence of a well-defined lesion margin and pleural traction. The average pair-wise percentage agreement was relatively high in nodule type and pleural retraction (81% and 89% respectively). A considerably lower agreement, however, was found for features that had to be rated qualitatively such as lesion margin in general or the subcategory of a well-defined lesion margin (63% and 47%, respectively), indicating that these features do not appear to be sufficiently definable by visual analysis to serve as a broadly applicable criterion within a screening process.

Interestingly however, when looking at the subcategory of a well-defined border, three observers scored linearly demarcated border more frequently in transient lesions (27/40, 18/27, and 23/39) compared to persistent lesions. We did not prospectively define whether the linear demarcation following the lobular border had to be present in several projections, which most likely contributed to the fact that one observer scored the feature only 4 times. The finding of linear demarcation shows similarity with a finding reported by Felix et al. [13]. Their study described a polygonal shape (defined “as a lesion with linear or concave margins at every corner”) as indicative for a transient lesion. Furthermore, they found that transient subsolid nodules were more frequently lobulated than persistent nodules. The finding of lobulation being predictive for transience reported by Felix et al. [13] is in contradiction to the other study by Lee SM et al. [19], who reported lobulation as indicative for malignancy. Similarly we found that 74% to 100% of the lobulated lesions were found to be persistent (15/16, 28/36, 1/1, 20/27 respectively).

In this study we selected the subsolid nodules following the nodule type annotations of the NLST database. Previous studies have shown that the agreement among radiologists is only moderate with regards to the differentiation of part-solid, non-solid and solid nodules [20, 21]. Therefore we decided to exclude all lesions that were considered not a subsolid nodule by at least 2 of the 4 experienced observers in our study, as indicated in their comments. We did so, to increase accuracy and reliability of the observer data.

Our study has some limitations. First, our study did not include any elaborate texture or quantitative analysis. Visual CT features in combination with elaborate objectively quantifiable measures might not only improve performance but also achieve a higher reproducibility. Second, we selected lesions ≥ 10 mm only, taking into account the fact that the majority of the NLST CTs has not been reconstructed with 1 mm slice thickness, thus not providing isotropic high resolution image quality in all three projections. The level of performance and reader agreement we found, confirms the notion that visual assessment of morphological features in lesions < 10mm will be even more difficult and less reliable. Last, the CT examinations of the NLST trial have been obtained with different scanners and variable slice thickness. Though only scans with a slice thickness of ≤ 2 mm were included, the diverging image quality might have influenced the visual assessment of the nodules.

In conclusion, experienced radiologists are moderately able to determine persistent and transient nodule character in lesions ≥ 10 mm visually. There are morphological features indicative for the discrimination of persistent and transient nodules, but none of them yielded significant discrimination in all four observers. Our results show that performance of the visual assessment of CT morphology alone is not sufficient to generally abandon a short-term follow-up and inter-reader variability plays a substantial role even among highly experienced observers.

## Supporting information

S1 File Main data file. Data file containing observers’ scores (probability that the lesion was persistent on a scale between 0–100 and morphology) and the ground truth for each case.
(SAV)
